# Insulin and TLR4 Inhibitor Improve Motor Impairments in a Rat Model of Parkinson’s Disease

**DOI:** 10.5812/ijpr-144200

**Published:** 2024-06-11

**Authors:** Fatemeh Hemmati, Neda Valian, Abolhassan Ahmadiani, Zahurin Mohamed, Raymond Azman Ali, Norlinah Mohamed Ibrahim, Seyed Farshad Hosseini Shirazi

**Affiliations:** 1 Pharmaceutical Sciences Research Center, Shahid Beheshti University of Medical Sciences, Tehran, Iran; 2 Neuroscience Research Center, Shahid Beheshti University of Medical Sciences, Tehran, Iran; 3 Department of Pharmacology, Faculty of Medicine, University of Malaya, 50603, Kuala Lumpur, Malaysia; 4 Department of Medicine, University Kebangsaan Malaysia Medical Centre, Cheras, Kuala Lumpur, Malaysia; 5 Department of Toxicology and Pharmacology, School of Pharmacy, Shahid Beheshti University of Medical Sciences, Tehran, Iran

**Keywords:** Parkinson’s Disease, Insulin, Insulin Resistance, TLR4, Intranasal Administration, S961, TAK242

## Abstract

**Background:**

Insulin resistance is an important pathological hallmark of Parkinson’s disease (PD). Proinflammatory cytokines during neuroinflammation decrease insulin sensitivity by suppressing insulin signaling elements. Toll-like receptor 4 (TLR4), the main receptor involved in neuroinflammation, is also associated with the pathogenesis of PD.

**Objectives:**

The present study evaluated the effect of insulin, an insulin receptor antagonist, and a TLR4 inhibitor on behavioral deficits and insulin resistance induced by 6-hydroxydopamine (6-OHDA).

**Methods:**

Male Wistar rats were divided into nine groups: (1) sham (normal saline [NS] in the medial forebrain bundle [MFB]); (2) 6-OHDA (20 µg in the MFB); (3) 6-OHDA + NS; (4) 6-OHDA + dimethyl sulfoxide (DMSO); (5) 6-OHDA + insulin (2.5 IU/day, intracerebroventricular ([ICV]); (6) 6-OHDA + insulin (5 IU/day, intranasal [IN]); (7) 6-OHDA + insulin receptor antagonist (S961; 6.5 nM/kg, ICV); (8) 6-OHDA + TLR4 inhibitor (TAK242; 0.01 µg/rat, ICV); (9) 6-OHDA + insulin + TLR4 inhibitor. All treatments were administered for seven consecutive days. Motor performance was evaluated using apomorphine-induced rotation and cylinder tests. Gene expression and protein levels of α-synuclein, TLR4, insulin receptor substrate (IRS) 1, IRS2, and glycogen synthase kinase 3β (GSK3β) were measured by real-time PCR and western blotting, respectively, in the striatum.

**Results:**

Insulin, alone and with TAK242, improved motor deficits induced by 6-OHDA. Administration of the insulin receptor antagonist had no effect on motor deficits. The increased expression of α-synuclein and TLR4 following 6-OHDA was attenuated by insulin and TAK242. GSK3β levels, both mRNA and protein, were significantly increased by 6-OHDA and attenuated with insulin and TAK242.

**Conclusions:**

The findings suggest that 6-OHDA induces neurodegeneration via activation of TLR4 and GSK3β, indicating insulin resistance, and that insulin can improve these impairments. Moreover, TLR4 inhibition prevents insulin signaling dysfunction and improves behavioral and molecular impairments, highlighting the critical role of TLR4 in the development of insulin resistance in PD pathology.

## 1. Background

Parkinson’s disease (PD), the second most common neurological disorder after Alzheimer's disease, is mainly characterized by the loss of dopaminergic neurons in the midbrain (1). Dopamine depletion in the nigrostriatal pathway leads to motor symptoms including bradykinesia, resting tremor, rigidity, and postural instability. There is growing evidence that insulin resistance and diabetes, which share similar pathological pathways, are important contributors to PD (2).

For many years, insulin was thought to act merely as a peripheral hormone involved in glucose homeostasis and energy metabolism. Nowadays, there is strong evidence that insulin affects several functions in the brain, such as mitochondrial function, gene expression, protein synthesis, and synaptic transmission through PI3K/Akt and MAPK/ERK pathways (3-5). Insulin receptors are expressed in different parts of the brain including the striatum and substantia nigra, and disruption of insulin signaling causes dopaminergic neuron dysfunction and death (6). Insulin resistance is defined as insulin desensitization, characterized by a decrease in the level and function of insulin signaling pathway elements (5). Many studies have shown the relationship between insulin resistance in the brain and neurodegenerative diseases like PD (5, 7). Increased risk of development of PD in diabetic patients (8), and the positive effects of anti-diabetic medications in patients with PD (9), have been reported in several studies. Central insulin resistance is mostly induced by chronic neuroinflammation in the brain and can promote neuronal cell death (5). These findings suggest that insulin signaling pathways can be considered a potential target for PD modification.

Toll-like receptors (TLRs), especially TLR2 and toll-like receptor 4 (TLR4), are involved in innate immune responses through the production of pro-inflammatory cytokines (10). Overactivation of TLR4 during chronic neuroinflammation reduces insulin sensitivity and causes insulin resistance (11-13), directly through pro-inflammatory kinases and reactive oxygen species, or indirectly through the release of pro-inflammatory cytokines and insulin-desensitizing factors (14). Toll-like receptor 4 is highly expressed in the substantia nigra and putamen of human brains (15) and plays an important role in the pathogenesis of PD (16, 17). Experimental evidence indicates that the inflammatory response induced by α-synuclein is mediated by TLR4 (18), and 1-methyl-4-phenyl-1,2,3,6-tetrahydropyridine (MPTP)-induced PD pathology is less severe in TLR4 knockout mice than in wild-type mice (15).

## 2. Objectives

Given the involvement of insulin receptor signaling and TLR4 in PD pathology, the present study evaluated the effect of modulation of these pathways using insulin, S961, and TAK242 on motor performance in a rat model of PD induced by 6-hydroxydopamine (6-OHDA). Additionally, insulin signaling pathway elements, as markers of insulin resistance, were assessed.

## 3. Methods

### 3.1. Animals

Male Wistar rats (220 - 250 g) were housed five per cage with food and water available ad libitum, under a standard 12-hour light/dark cycle and a temperature of 23 ± 2ºC. The rats were acclimated to the animal colonies for 5 to 6 days. This study was approved by the Animal Research Ethics Committee of Shahid Beheshti University of Medical Sciences (IR.SBMU.RETECH.REC.1400.676).

### 3.2. Stereotaxic Surgery

The rats were placed on a stereotaxic instrument (Stoelting, USA) after being anesthetized with an intraperitoneal injection of ketamine/xylazine (80/20 mg/kg). A total of 20 µg of 6-OHDA (H4381; Sigma, USA), dissolved in 2 µL of sterile 0.9% normal saline (NS) with 0.2 mg/mL ascorbic acid, was injected over 5 minutes into the right medial forebrain bundle (MFB) (AP: -5.2; ML: +2.4; DV: 8.4) using a Hamilton syringe (22 µL). The control rats received the same volume of vehicle (2 µL of sterile 0.9% NS with 0.2 mg/mL ascorbic acid). A guide cannula was placed in the right lateral ventricle (AP: -0.75, ML: +1.7, DV: 4) for the daily injection of insulin, insulin antagonist, and TLR4 inhibitor. Normal saline and dimethyl sulfoxide (DMSO) (1%) were injected as vehicles.

### 3.3. Drugs Administrations

The rats were divided into nine groups (n = 10 per group) as follows:

(1) Sham: NS with 0.2 mg/mL ascorbic acid in the right MFB

(2) 6-OHDA: 6-OHDA in the right MFB

(3) 6-OHDA + NS: 6-OHDA in the right MFB + NS (intracerebroventricular [ICV])

(4) 6-OHDA + DMSO: 6-OHDA in the right MFB + DMSO (ICV)

(5) 6-OHDA + insulin (ICV): 6-OHDA in the right MFB + insulin (Novo Nordisk Pharma, Bagsvӕrd, Denmark; 2.5 IU/day, ICV)

(6) 6-OHDA + insulin (IN): 6-OHDA in the right MFB + insulin (Novo Nordisk Pharma, Bagsvӕrd, Denmark; 5 IU/day, intranasal [IN])

(7) 6-OHDA + S961: 6-OHDA in the right MFB + S961 (NNC0069-0961; Novo Nordisk, Denmark) (6.5 nM/kg, ICV)

(8) 6-OHDA + TAK242: 6-OHDA in the right MFB + TAK242 (CLI-095; InvivoGen, USA) (0.01 μg/rat, ICV)

(9) 6-OHDA + insulin + TAK242: 6-OHDA in the right MFB + insulin (2.5 IU/day, ICV) + TAK242 (0.01 μg/rat, ICV)

**Figure 1.**
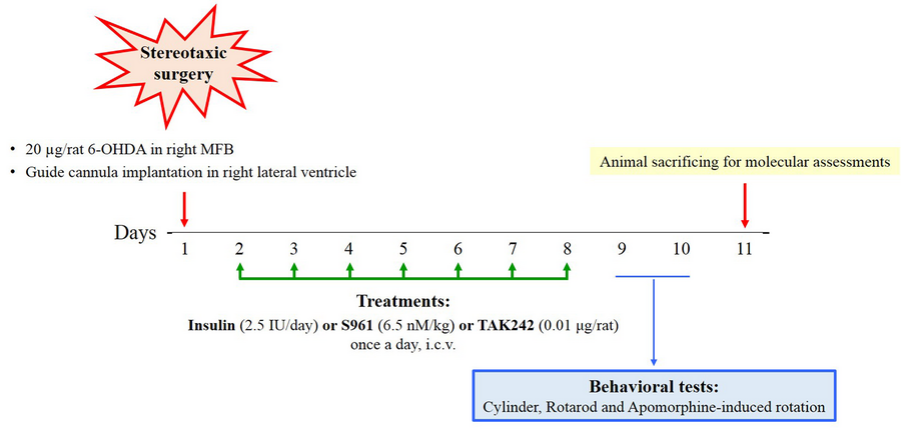
Experimental time-line

All treatments were continued for seven consecutive days. After the last injection of treatments, behavioral tests were performed, and then the animals were sacrificed for molecular assessments (n = 3 for real-time PCR and western blotting). The timeline of the experiments is shown in Figure 1. 

### 3.4. Behavioral Tests

#### 3.4.1. Apomorphine-induced Rotation Test

The animals were placed in the box for 20 minutes for habituation and then received a subcutaneous injection of apomorphine (0.5 mg/kg), dissolved in NS with 0.2 mg/mL ascorbic acid (19). The net contralateral rotations (contralateral - ipsilateral) were calculated over 40 minutes.

#### 3.4.2. Cylinder Test

The cylinder test is commonly used to assess motor-sensory function in animal models of PD (20). The asymmetric use of the forelimbs is due to a lesion in the nigrostriatal pathway, while normal rats use both forepaws equally (21). The rats were placed in a glass cylinder, and each touch on the cylinder wall using the right, left, or both front paws was counted. The results are expressed as the percentage of touches made with the contralateral paw to the total number of touches in 300 seconds.

### 3.5. RNA Isolation and qPCR Protocol

Total RNA from the right striatum was extracted using the Total RNA Isolation System (Qiagen, USA), according to the manufacturer's instructions. RNA concentration and purity (the ratio of absorbance values at 260 and 280 nm) were evaluated using a Nanodrop™ spectrophotometer (Nanodrop; Thermo Fisher Scientific, Wilmington, DE, USA). Then, cDNA was synthesized from 1 μg of total RNA using the RevertAid™ First Strand cDNA Synthesis Kit (Qiagen, USA) in accordance with the manufacturer's protocols, and used to quantitatively measure the expression of genes using SYBR Green Real-Time PCR Master Mix reagents in the ABI system (USA). Relative expression of α-synuclein, TLR4, insulin receptor substrate (IRS) 1, IRS2, and glycogen synthase kinase 3β (GSK3β) was calculated by the 2^-ΔΔCT^ method, with β-actin used as an internal control. Primer sequences used for qPCR are presented in Table 1. 

**Table 1.** Primer Sequences Use for qPCR

Gene	Forward Primers (5′3′)	Revers Primers (5′3′)
**α-synuclein**	CCAACATATAGGCTGGAGTG	TAGCCATCCACAGACACACC
**TLR4**	GTGGGTCAAGGACCAGAAAA	GGCTACCACAAGCACACTGA
**IRS1**	AGGTTTTCCCCTCCTAGCAA	GCTGAGATCGAAACATGCAA
**IRS2**	GGCTCACCAGTTTTCTGCTC	GTAGAATTGCTCCCGTTGGA
**GSK3β**	TCGGCTCTCTCCTTCCATTA	CCCTCATCCCTGTACCTCAA
**β-actin**	TAGGGTCCATTGGTGGAAAC	TGCCGATAGTGATGACCTGA

### 3.6. Western Blotting

A Potter-Elvehjem tissue grinder (Sigma, St. Louis, Missouri, USA) with chilled tris-buffered saline with tween (TBST) (20 mM tris, pH 7.5; 0.75 M NaCl; 2 mM 2-mercaptoethanol) and 10 µL/mL protease inhibitor cocktail (Sigma) was used to homogenize striatum samples, which were then centrifuged at 23,000 g at 4°C for 45 minutes. The protein concentrations of the supernatant were quantified using a bicinchoninic acid protein assay kit (Sigma-Aldrich) with bovine serum albumin (BSA) as the standard. Next, 25 µg of protein was loaded onto sodium dodecyl sulfate-polyacrylamide gels for electrophoresis. The separated proteins were transferred to polyvinylidene difluoride membranes (MSI, Westborough, Massachusetts, USA), and non-specific binding sites were blocked using blocking buffer (TBST + BSA) for 1 hour. The membranes were incubated with rabbit anti-TLR4 (1:1000, ab22048, Abcam), anti-α-synuclein (1:1000, ab52168, Abcam), anti-IRS1 (1:1000, ab52167, Abcam), anti-IRS2 (1:1000, ab134101, Abcam), anti-GSK3β (1:1000, ab2602, Abcam), and anti-β-actin (1:1000, ab20272, Abcam) antibodies at 4°C overnight. After washing in TBST, the membranes were incubated with rabbit anti-mouse secondary antibody (1:2000, ab6728, Abcam) for 30 minutes at room temperature. The immunoreactive bands were visualized using an enhanced chemiluminescent substrate (ChemiGlow; Alpha Innotech, San Leandro, California, USA) and a chemiluminescent imaging system (FluorChem 5500; Alpha Innotech). Quantification of the bands' density was performed using Image J software.

### 3.7. Statistical Analysis

One-way analysis with Tukey’s post hoc tests was used for the statistical comparison of behavioral and molecular data, using the 16th version of SPSS. Data are reported as mean ± standard error of the mean (SEM), and P < 0.05 was considered statistically significant.

## 4. Results

### 4.1. Improvement of 6-OHDA-Induced Motor Impairment by Insulin

**Figure 2.**
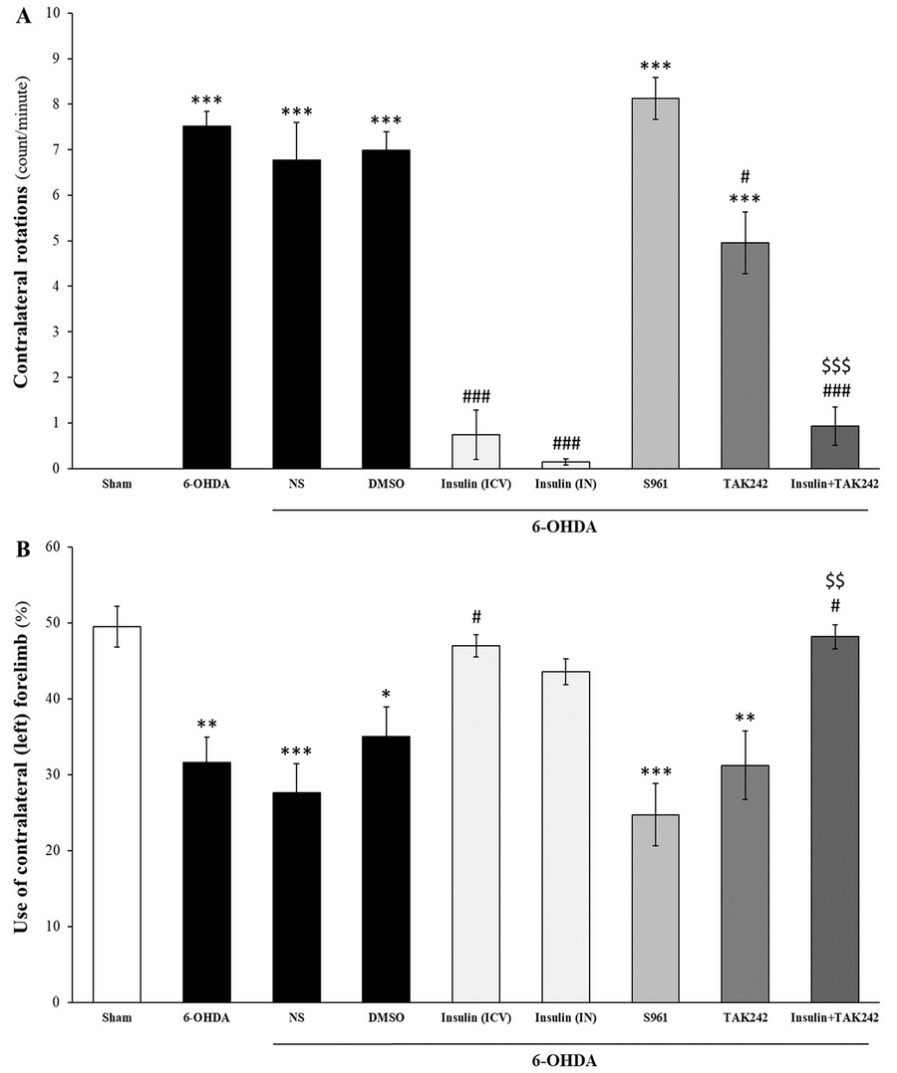
Insulin treatment improved motor impairments induced by 6-hydroxydopamine (6-OHDA). Administration of 6-OHDA induced motor deficits in apomorphine-induced rotation (A); and cylinder (B) tests. Insulin, both intracerebroventricular (ICV) and intranasal (IN), and TAK242 decreased contralateral rotations. Insulin combined with TAK242 was more effective than TAK242 alone (A); insulin (ICV) and combination therapy with TAK242 significantly increased the use of the contralateral hand, whereas S961 and TAK242 alone had no effects on forelimb asymmetry (B). Data are presented as mean ± standard error of the mean (SEM) (n = 10). * P < 0.05, ** P < 0.01, *** P < 0.001 vs. sham; # P < 0.05, ### P < 0.001 vs. 6-OHDA; $$ P < 0.01, $$$ P < 0.001 vs. 6-OHDA + TAK242.

Insulin, both IN and ICV, improved motor impairments induced by 6-OHDA (Figure 2). As shown in Figure 2A, 6-OHDA caused contralateral rotations induced by apomorphine compared to the sham group [F _(8, 81)_ = 52.268, P < 0.001]. Intranasal and ICV administrations of insulin significantly reduced contralateral rotations, both alone (P < 0.001) and in combination with TAK242 (P < 0.001). TAK242 also decreased contralateral rotations compared to 6-OHDA (P < 0.05), but there was a significant increase compared to the sham group (P < 0.001). Combination therapy of insulin and TAK242 was more effective than TAK242 alone in reducing apomorphine-induced rotation (P < 0.001). S961 had no effect on apomorphine-induced rotations (P < 0.001 compared to sham).

Findings from the cylinder test indicated that 6-OHDA decreased the use of the contralateral forelimb [F _(8, 81)_ = 8.817, P < 0.001], and insulin (ICV) significantly improved forelimb asymmetry by increasing the use of the lesioned hand (P < 0.05 vs. 6-OHDA). Intranasal insulin partially improved this motor deficit (P > 0.05 vs. sham). Although S961 and TAK242 alone did not improve this impairment, the combination treatment of insulin and TAK242 increased the use of the left hand compared to 6-OHDA (P < 0.05). The effect of insulin and TAK242 was better than TAK242 alone (P < 0.01) (Figure 2B). 

### 4.2. Insulin and TAK242 Attenuated α-Synuclein and TLR4 Gene Expression Following 6-OHDA

**Figure 3.**
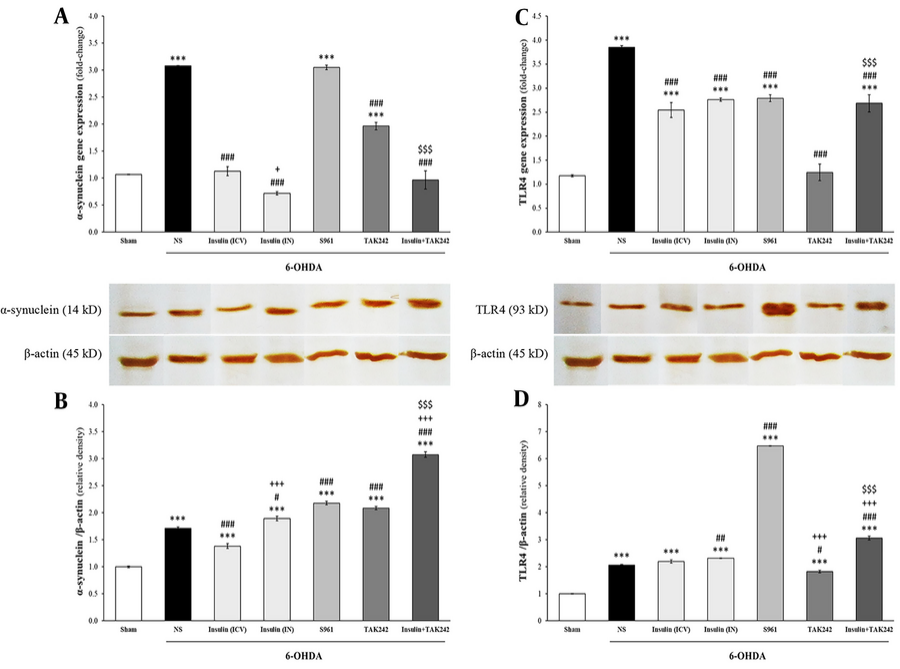
Insulin, S961 and TAK242 effects on the expression of α-synuclein and toll-like receptor 4 (TLR4). The mRNA and protein levels of α-synuclein (A, B); and TLR4 (C, D) were significantly increased by 6-hydroxydopamine (6-OHDA). Although α-synuclein mRNA was reduced by insulin, TAK242 and insulin + TAK242 (A); but its protein decreased just by intracerebroventricular (ICV) administration of insulin (B); all treatment decreased TLR4 gene expression (C); however, TLR4 protein only attenuated by TAK242. Data are expressed as mean ± standard error of the mean (SEM) (n = 3). *** P < 0.001 vs. sham; # P < 0.05, ## P < 0.01, ### P < 0.001 vs. 6-OHDA; + P < 0.05, +++ P < 0.001 vs. 6-OHDA + insulin (ICV); $$$ P < 0.001 vs. 6-OHDA + TAK242.

mRNA and protein levels of α-synuclein and TLR4 were assessed using qPCR and western blot (Figure 3). 6-hydroxydopamine significantly increased α-synuclein mRNA [F _(6, 14)_ = 161.992, P < 0.001] and protein [F _(6, 14)_ = 326.364, P < 0.001] (Figure 3A and B). Insulin, TAK242, and insulin + TAK242 reduced α-synuclein gene expression compared to 6-OHDA (P < 0.001). Intranasal insulin administration was more effective than ICV in decreasing α-synuclein mRNA (P < 0.05). α-Synuclein mRNA in animals treated with insulin + TAK242 was lower than in those treated with TAK242 alone (P < 0.001), suggesting the greater effectiveness of combination therapy. α-Synuclein gene expression was significantly increased in S961-treated animals compared to 6-OHDA (P < 0.001) (Figure 3A). α-Synuclein protein, which increased in the 6-OHDA group, was significantly attenuated following treatment with ICV administration of insulin (P < 0.001). Intranasal insulin, S961, TAK242, and insulin + TAK242 did not significantly decrease it (P < 0.001 and P > 0.05 compared to sham and 6-OHDA, respectively) (Figure 3B). 

Gene expression [F _(6, 14)_ = 66.311, P < 0.001] and protein [F _(6, 14)_ = 1808.634, P < 0.001] levels of TLR4 were enhanced following 6-OHDA injection (Figure 3C and D). All treatments reduced TLR4 mRNA compared to 6-OHDA (P < 0.001) (Figure 3C). Toll-like receptor 4 protein was only decreased by TAK242 (P < 0.05 vs. 6-OHDA) (Figure 3D). 

### 4.3. IRS1 and 2 Expression and Protein Elevated by Insulin

**Figure 4.**
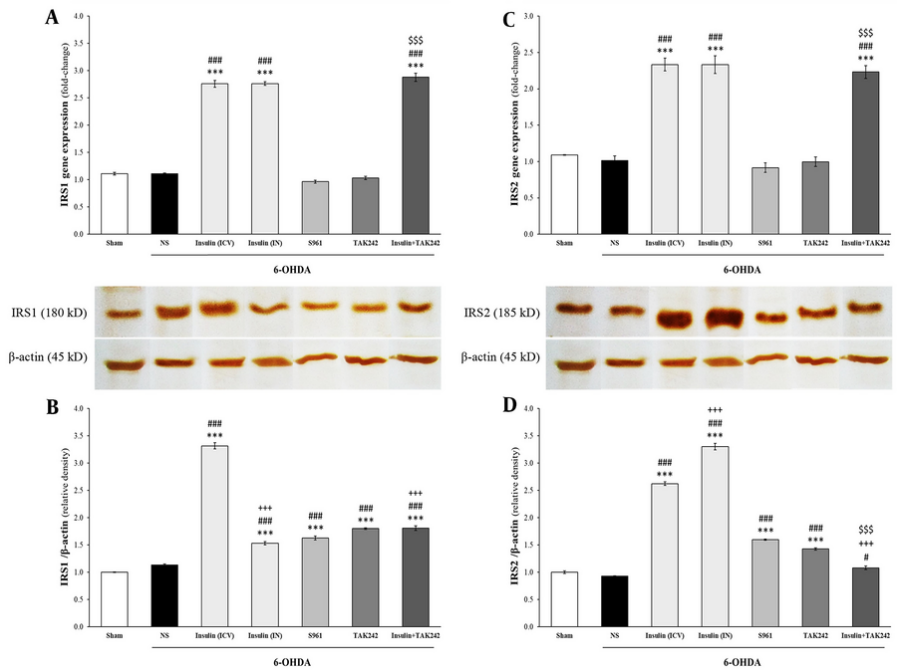
The effect of different treatment on insulin receptor substrate (IRS) 1 and 2. Injection of 6-hydroxydopamine (6-OHDA) had no effect on the mRNA and protein of IRS1 (A, B) and IRS2 (C, D). Insulin and insulin + TAK242 increased IRS1 and 2 expression, but S961 and TAK242 did not change them (A, C). IRS1 and 2 proteins were enhanced by insulin, S961 and TAK242, bun not insulin + TAK242 (B, D). Data are presented as mean ± standard error of the mean (SEM) (n = 3). *** P < 0.001 vs. sham; # P < 0.05, ### P < 0.001 vs. 6-OHDA; +++ P < 0.001 vs. 6-OHDA + insulin (intracerebroventricular [ICV]); $$$ P < 0.001 vs. 6-OHDA + TAK242.

Changes in the mRNA and protein levels of IRS1 and IRS2 are shown in Figure 4. Statistical analysis demonstrated that although 6-OHDA had no effect on IRS1 gene expression [F _(6, 14)_ = 453.446, P < 0.001] and protein levels [F _(6, 14)_ = 523.951, P < 0.001], insulin significantly increased both in comparison to sham and 6-OHDA groups (P < 0.001). Insulin + TAK242 also significantly increased IRS1 mRNA levels compared to sham, 6-OHDA, and TAK242 alone (P < 0.001). S961 and TAK242 did not change IRS1 mRNA levels (P > 0.05 compared to sham) (Figure 4A). Insulin receptor substrate 1 protein levels were enhanced by all treatments (P < 0.001 vs. sham and 6-OHDA). Intracerebroventricular administration of insulin was more effective than IN insulin and insulin + TAK242 in increasing IRS1 protein levels (P < 0.001) (Figure 4B). 

Similar to IRS1, IRS2 levels were not changed by 6-OHDA, but significantly increased following treatment with ICV and IN insulin at both mRNA [F _(6, 14)_ = 79.247, P < 0.001] and protein [F _(6, 14)_ = 812.855, P < 0.001] levels. Elevated IRS2 gene expression was also observed in the insulin + TAK242 group compared to sham, 6-OHDA, and TAK242 alone (P < 0.001) (Figure 4C). Insulin receptor substrate 2 protein levels were significantly increased by insulin (IN and ICV), S961, and TAK242 in comparison to sham and 6-OHDA groups (P < 0.001) (Figure 4D). 

### 4.4. Increased Levels of GSK3β Following 6-OHDA was Reduced by Insulin and TAK242

**Figure 5.**
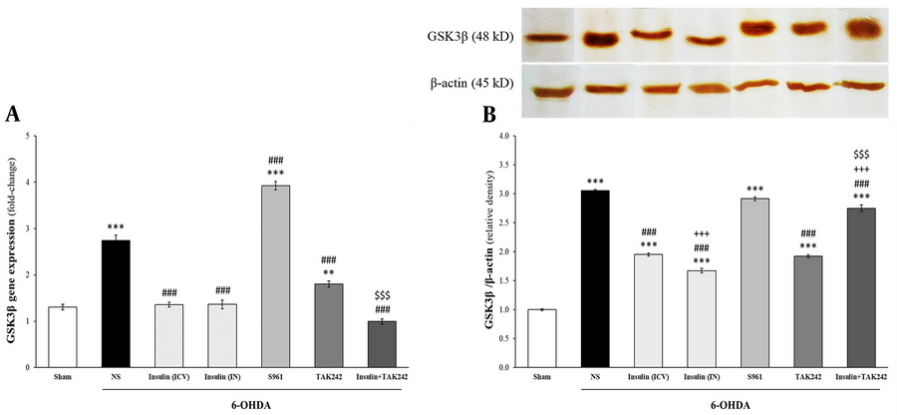
Insulin and TAK242 attenuated glycogen synthase kinase 3β (GSK3β) following 6-hydroxydopamine (6-OHDA). Gene expression (A); and protein (B) of GSK3β were enhanced by 6-OHDA. Insulin (intracerebroventricular [ICV] and intranasal [IN]), TAK242 and insulin + TAK242 reduced both of them. S961 had no effect on GSK3β. Data are reported as mean ± standard error of the mean (SEM) (n = 3). ** P < 0.01, *** P < 0.001 vs. sham; ### P < 0.001 vs. 6-OHDA; +++ P < 0.001 vs. 6-OHDA + insulin (ICV); $$$ P < 0.001 vs. 6-OHDA + TAK242.

Statistical analysis indicated that the levels of GSK3β mRNA [F _(6, 14)_ = 181.239, P < 0.001] and protein [F _(6, 14)_ = 516.394, P < 0.001] were significantly different between groups (Figure 5). The mRNA of GSK3β was elevated by 6-OHDA (P < 0.001), while insulin (both IN and ICV), TAK242, and insulin+TAK242 significantly decreased it in comparison to 6-OHDA (P < 0.001). Insulin+TAK242 reduced GSK3β gene expression more than TAK242 alone (P < 0.001). In animals treated with S961, GSK3β expression was not only not reduced but was also significantly higher than in the 6-OHDA group (P < 0.001) (Figure 5A). 

6-hydroxydopamine also increased GSK3β protein levels (P < 0.001). Insulin, TAK242, and insulin + TAK242 attenuated this increase in comparison to 6-OHDA (P < 0.001); however, GSK3β protein levels were still higher in these groups than in the sham group (P < 0.001). S961 had no effect on GSK3β protein levels compared to 6-OHDA (P < 0.001) (Figure 5B). 

## 5. Discussion

These findings revealed that 6-OHDA induced motor impairments by increasing the expression of α-synuclein and TLR4 in the striatum. It also elevated GSK3β, an indicator of insulin resistance, at both mRNA and protein levels. Administration of insulin (IN and ICV) and TLR4 inhibitor (TAK242) attenuated these toxic effects, leading to improvements in behavioral deficits. However, suppression of the insulin signaling pathway by S961 prevented the positive effects of insulin.

Insulin regulates dopamine synthesis and release by modulating the expression of tyrosine hydroxylase (TH), the rate-limiting enzyme in dopamine synthesis (22). Central insulin resistance during aging may result in dopaminergic neuron dysfunction (5, 23). Reduced insulin receptor mRNA and immunoreactivity, along with lower levels of TH, have been previously observed in the substantia nigra pars compacta of patients with PD (24, 25). Therefore, activation of insulin signaling pathways may protect against 6-OHDA-induced toxicity in dopaminergic neurons. In this regard, an experimental study has shown that IN insulin administration protects dopaminergic neurons against cell death in the 6-OHDA model of PD and attenuates motor impairments (26).

α-Synuclein is the main component of Lewy bodies and Lewy neurites observed in the brains of PD patients (27). It disrupts mitochondrial function via interaction with complex I in the respiratory chain (28). α-Synuclein is a presynaptic protein involved in neurotransmitter release, synaptic transmission, and mitochondrial function (27). It also acts as an immune signaling molecule that activates microglia and astrocytes through TLR4 (15, 16, 29, 30).

In the present study, we observed increased gene expression of α-synuclein concomitant with elevated TLR4 expression and motor deficits. In parallel with these findings, it has been previously shown that overexpression of α-synuclein leads to motor dysfunction in mice via microglial overactivation and increased TLR4 expression. Furthermore, suppression of microglial activation improved motor deficits (18). In vitro studies have also shown that lithium and IGF-1 reduce astrocyte activation and inflammatory factors following LPS treatment through inhibition of TLR4 expression (31, 32). Our findings revealed that IN and ICV insulin administration improved motor impairments induced by 6-OHDA, at least in part, via the reduction in α-synuclein and TLR4 gene expression.

Insulin plays a critical role in autophagy regulation through the PI3K/Akt/mTOR pathway, ultimately leading to autophagy activation (33). Pharmacological inhibition of mTORC1 by rapamycin has been shown to decrease α-synuclein aggregation (34) and prevent dopaminergic neuron loss (35). In the present study, increased IRS1 and IRS2 levels in animals receiving insulin confirmed the activation of the insulin signaling pathway. Additionally, the reduced protein level of α-synuclein indicated that insulin administration activated the autophagy process, thereby reducing α-synuclein levels. Consistent with our findings, another study also revealed that insulin reduced α-synuclein in PC12 cells following treatment with MPP+ (36).

Toll-like receptor 4 is elevated in the blood and brain of patients with PD and in animal models of PD (18, 37, 38). The severity and duration of the disease, as well as responsiveness to PD medication, are strongly correlated with TLR4 levels in the blood (38), suggesting that TLR4 modulation could be a promising approach to improve PD pathology. In line with clinical evidence, experimental studies have reported the neuroprotective effect of TLR4 knockout against MPTP neurotoxicity in animal models of PD (39, 40). Reduced expression of TLR4 and MyD88, important downstream elements of the TLR4 signaling pathway, by rosmarinic acid has been shown to improve motor impairment by decreasing α-synuclein and preventing TH^+^ neuron degeneration in the MPTP mouse model of PD (41). Here, in line with the evidence mentioned above, we also demonstrated that TLR4 inhibition by TAK242 decreased the expression of α-synuclein and TLR4, thereby significantly improving behavioral impairments following 6-OHDA injection in rats. TAK242 had better effects when co-administered with insulin.

Several epidemiological and experimental studies have revealed the association between diabetes and PD (8, 9). Insulin resistance in patients with type 2 diabetes leads to dopaminergic neuron degeneration (42), suggesting that diabetic patients are more susceptible to PD and experience more severe movement symptoms compared to non-diabetic PD patients (8, 43, 44). Moreover, the neuroprotective effect of anti-diabetic medications in patients with PD (9, 45-48) confirms the importance of insulin signaling and insulin resistance in PD.

Proinflammatory cytokine production during chronic inflammation in neurodegenerative diseases can induce insulin resistance through IRS serine phosphorylation, which decreases IRS interaction with the insulin receptor (49, 50). Increased levels and activity of GSK3β have been observed in insulin resistance and other pathological conditions (50). Glycogen synthase kinase 3β, a cellular serine/threonine protein kinase, plays critical roles in several processes like cell division, differentiation, proliferation, cellular adhesion, neuronal plasticity and polarity, synaptic function, and neurotransmitter release (51). It is expressed throughout the brain, and its activity is negatively regulated by phosphorylation on Ser9 by pro-survival signaling pathways like insulin. However, phosphorylation on Tyr216 increases GSK3β activity (23). The insulin signaling pathway regulates GSK3β activation via the IRS/PI3K/Akt pathway (52).

In the present study, we observed an elevation in gene expression and protein levels of GSK3β – as a marker of insulin resistance – following 6-OHDA injection. In parallel with this finding, postmortem studies have previously found an increase in p-Tyr216-GSK3β and α-synuclein expression in the striatum of PD patients (53, 54). We also indicated that IN and ICV insulin administration activated the insulin signaling pathway, leading to decreased GSK3β at both mRNA and protein levels.

### 5.1. Conclusions

In general, the findings of the present study indicated that insulin signaling was impaired in the 6-OHDA rat model of PD, accompanied by overexpression of TLR4 and α-synuclein, which led to motor deficits. Insulin, both IN and ICV, and the TLR4 inhibitor (TAK242) improved these impairments. Co-treatment with insulin and TAK242 had a better effect, suggesting that combination therapy may be an effective therapeutic approach to attenuate PD pathology. However, more studies are needed to elucidate the molecular interaction between insulin and TLR4 signaling pathways.

## Data Availability

The dataset presented in the study is available on request from the corresponding author during submission or after publication.
